# William Harvey

**Published:** 1898-06

**Authors:** 


### Reviews of Books.

---

**William Harvey.** By D'ARCY POWER. Pp. xi., 283. London: T. Fisher Unwin. 1897.

This is one of the "Masters of Medicine" series, the introductory volume of which we reviewed in March. Everything has been done to make the record of the life of this truly great man as complete as possible; and although the material at the author's disposal was somewhat scanty, yet we have a consecutive and very readable biography supplemented by well-chosen extracts from Harvey's works. The authorities are enumerated in an appendix and there is a good index.

Our admiration for the indomitable energy and patience which led to the discovery and promulgation of the method of the circulation of the blood is much increased by this book; and the grace and tact which made Harvey so good a courtier, as well as the skill and learning which made him so eminent a physician, are well brought out.

It is interesting to note the strict conditions by which a physician to St. Bartholomew's Hospital was bound early in the seventeenth century; and the charge read to the applicant for such a post is full of solemnity. Whatever we may think of our profession now, it is certain that it was in those days considered a high one, and the duties of the hospital staff were honoured and sacred. It is needless to say that Harvey fulfilled these conditions during his tenure of office. He was a good Latin scholar, and made his notes in that language or in English, as if he thought equally well in either; and this sometimes led to comical results, such as the following example: "Exempto corde, frogg scipp, eele crawle, dogg Ambulat"; truly an instance of "dog Latin."

As is well known, Harvey's doctrines were strenuously opposed by many, and it required very firm convictions to enable a man to persevere as he did. His struggles to make his researches known are admirably told by Mr. D'Arcy Power. Everyone who is interested in the heroes of medicine should study this book.

